# Transportation of Cull Sows—Deterioration of Clinical Condition From Departure and Until Arrival at the Slaughter Plant

**DOI:** 10.3389/fvets.2019.00028

**Published:** 2019-02-18

**Authors:** Karen Thodberg, Katrine Kop Fogsgaard, Mette S. Herskin

**Affiliations:** Department of Animal Science, AU Foulum, Aarhus University, Aarhus, Denmark

**Keywords:** cull sow, transportation, fitness for transport, pre-slaughter logistic chain, welfare

## Abstract

Cull sows may be more vulnerable to transportation compared to other swine categories, as they are typically culled after several production cycles, and hence may be injured or weak. Until now, transportation of sows has received very little scientific attention. We aimed to investigate whether the clinical condition of the sows changed during transportation from commercial Danish farms to slaughter plants, and to initiate identification of potential risk factors for such deterioration. This observational study included 522 sows in 47 batches from 12 farms, varying according to transportation time from farm to slaughter plant. Standardized clinical examinations were conducted on-farm and on the slaughter plant. In addition, data on transportation duration, number and duration of stops, temperature during driving, and during waiting before unloading were collected. The sows' median parity was five (range 1–11) and close to 40% were lactating at the day of transportation. The mean duration of transportation was 232 ± 113 min, and the mean temperature in the trucks was 14.1 ± 5.3°C. Half of the clinical variables recorded before and after transportation changed significantly. Among these were injuries (e.g., superficial skin lesions, totally, *P* < 0.000; front, *P* < 0.001; wounds, *P* < 0.001; gait score, *P* < 0.001), and measures possibly related to heat stress (e.g. skin elasticity, *P* < 0.001). Three sows arrived in a condition as legally unfit for transport. The deterioration of the sows' condition was mainly related to transportation factors, such as temperature and duration—often in interaction—as well as duration of stops during the journey and while waiting before unloading. The changes in clinical condition were less dependent on the pre-transportation clinical condition of the sows, such as parity, body condition score and gait score. The results show that the clinical condition of the cull sows deteriorated from farm to slaughter plant, thereby adding data to the debate on fitness for transport of cull sows. The main risk factors were not related to characteristics of the sows, but of the journeys. Future studies should focus on identifying and distinguishing between risk factors in order to develop procedures that allow transportation of cull sows to slaughter without jeopardizing their welfare.

## Introduction

International pig production is characterized by increasing herd sizes and changes in the slaughter industry toward fewer and larger units [as discussed by (1)] leading to increasing transportation distances from farm to slaughter. Pig transportation is a multifactorial stressor (2), but until now studies have focused almost exclusively on market weight pigs (3–5).

Cull sows are a distinct category of swine and may be more vulnerable to transportation stress than market weight pigs (6, 7). Sows are typically culled after several production cycles, and hence may be injured or weak (8, 9). For this group of animals, OIE (10) recommends special conditions on the day of slaughter in order to ensure animal welfare. Recently, we described the clinical condition of cull sows on-farm and found parities ranging from 1 to 11, and shoulder ulcers in 10% of the examined animals and vulva lesions in 7% of the sows (11).

The clinical condition of cull sows has been reported upon arrival at slaughter plants (12, 13) and McGee et al. (14) found that cull sows—compared to market weight pigs—made up the majority of swine arriving at U.S. buying stations as fatigued, lame or in a very low body condition. In addition, Malena et al. (15), Lykke et al. (16) and Peterson et al. (17) found increased mortality in sows upon arrival at slaughter plants as compared to other groups of swine. However, none of these recorded the clinical condition of the sows before transportation, and hence it cannot be determined whether the conditions observed at unloading were merely a reflection of the on-farm clinical conditions or the result of changes occurring during transportation. According to European legislation (18), injured farm animals cannot be transported, whereas slightly injured animals may be fit for transportation. However, for all animals, transportation may only take place under conditions that are not leading to injury or unnecessary suffering. Hence, in order to examine whether transportation of cull sows leads to a deterioration of their clinical condition, studies involving clinical examination of sows before and after transportation are needed. Such knowledge will be highly relevant for the assessment of fitness for transport.

One factor potentially contributing to the vulnerability of cull sows to transportation is the increased productivity of modern sows, characterized by high litter sizes and a large genetic potential for milk production, which might render the sows more sensitive to heat stress than just a few decades ago (19). Adding to this risk is the fact that a large proportion of sows are culled right after weaning (20, 21), where milk production is peaking (22). Examination of sow heat production shows a steady increase from farrowing to weaning (19). Recently, this knowledge has led to increased focus on the susceptibility of lactating sows to heat stress [e.g., (23–25)]. However, despite the large proportion of lactating animals among the population of cull sows, no studies have focused on their susceptibility to heat stress or examined whether cull sows, led almost directly from the farrowing barn and into the trucks transporting them to slaughter, are more vulnerable during transportation and to other stressful aspects of the pre-slaughter logistic chain.

The aim of the present study was to investigate whether the clinical condition of cull sows from commercial Danish sow farms change during transportation. In addition, data was collected to initiate the identification of selected risk factors for such deterioration—both at the sow level and related to the transportation as such (focusing on temperature in the truck and the duration and stops during transportation).

According to Danish legislation, which is stricter compared to European regulations (18), cull sows cannot be transported by commercial trucks for more than 8 h. This study comprised 522 sows transported as a subset of 47 full loads of sows and compared the clinical condition of the animals before and after transportation to slaughter. Fogsgaard et al. (11) have reported data on the pre-transportation clinical condition of the sows and Herskin et al. (26) reported data on the behavior of a sub-sample of the sows while waiting in stationary transfer vehicles before the commercial transportation.

## Materials and Methods

### Study Design and Participating Farms

This observational study included 47 batches of sows from 12 farms and involved a total of 522 sows. All farms participated voluntarily after being informed about the aim and procedures of the study. The farms were located in the western part of Denmark (Jutland and Funen) and delivered cull sows to a large slaughter plant (Danish Crown, Randers, Denmark) situated in the southern part of Denmark. Based on information from Danish Crown, the farms were divided into four categories, according to an estimated transportation time from farm to the slaughter plant of either 0–2; 2–4; 4–6, or 6–8 h, respectively. For details about recruitment of farms, see Fogsgaard et al. (11). After agreeing to participate in the study, we invited the farms' regular haulers to participate as well, and the seven haulers in question all accepted to take part in the data collection.

### Selection of Cull Sow Loads

The study period ran from January 2015 to February 2016, and the transportation of the cull sow loads selected for data collection were distributed throughout this period. With help from Danish Crown (Danish Crown, Randers, Denmark) coordinating logistics with farm owners, haulers and the slaughter plant, we selected sow loads for the study.

The selection of cull sows in the individual farms was done by the farm staff. According to an ethical approval from the Danish Animal Experiments Inspectorate (License number 2014-15-0201-00172) we were exempted from following the aggravated Danish requirements for fitness for transportation of sows, and to meet only the European Council Regulations (18). This meant that only sows that were clearly unfit for transport were excluded from the study. The excluded animals comprised sows that were unable support themselves on all four legs; sows with a prolapse or open wounds with a diameter of more than 5 cm; and sows that had completed at least 90% of their pregnancy; or given birth within the last week. On average 11.1 ± 2.8 experimental sows were selected for each load.

### Farm Information

Before the arrival of the university technicians, the staff at each farm filled in a list of information regarding the cull sows to be transported on that specific day, comprising information on parity, suggested culling reason, reproductive stage, housing in the period since the decision to cull was taken, and feed intake during the last 24 h before transportation (Table 1).

**Table 1:** Information provided by the 12 farms involved in the study about the 522 cull sows.

**Parameter**		**Category**	**Prevalence**
Housing (% sows)	Crated/loose	Crated	51.3
		Loose	8.8
		NA	39.8
	Single/group	Single	56.9
		Group	4.8
		NA	38.3
Culling reason (% sows)		Reproduction	24.5
		Age	22.2
		Health (physical problems)	10.9
		Other	4.8
		NA	37.5
Parity		Median	5
		IQR	[2, 3]
		Range	(1–11)
		Parity 1	3.3
		Parity > 6	27.8
		NA	29.9
Feed intake the last 24 h (% sows)		Yes	60.3
		No	0
		NA	39.7

*Missing data (data not available, NA) are presented as percentage of sows. IQR: Interquartile range*.

### Clinical Examinations Before and After Transportation

Two technicians arrived at the farm ~ 2–4 h before cull sows were transferred to a mobile pick-up facility or an on-farm pick-up facility. The trained technicians conducted a standard clinical examination of the sows, consisting of three parts done at (1) a distance of 1–2 m from the sow, before touching her; (2) close to the sow, while she was standing; and (3) while the sow was walking to the pick-up facility (gait score). In both the farm and later at the slaughter plant, we scored the gait in a straight corridor on concrete floor. The sows were typically moving forward voluntarily or otherwise driven gently forward without the use of physical force. A short description of the measures are shown in Tables 2, 3, and in Fogsgaard et al. (11).

**Table 2:** List of clinical measures recorded from 522 cull sows before and after transportation to the slaughter plant, where no significant changes were found between the two clinical examinations.

**Clinical measure**	**Description^**a**^**	**Unit or category**	**Farm**	**Slaughter plant**	**Statistics**
General condition (% sows)	Deviance from the normal condition (e.g., apathy, abnormal movement, excitement, coughing) was scored as “bad,” else “good”	Good	97.3	87.9	*P* = 0.13^b^
		Bad	1.0	0	
		NA	1.7	12.1	
Body temperature (°C)		Mean ± SD	37.9 ± 0.7	37.9 ± 0.8	*P* = 0.55
		NA	1.9	5.6	
Skin color (% sows)	The general color of the sow's skin	Normal	97.9	95.8	*P* = 0.18
		Pale	0.8	0.2	
		Red	0.4	0.2	
		NA	1.0	3.8	
Color of mucosa (% sows)	Note: Bluish mucosa was an option but was never observed	Normal	95.4	92.0	*P* = 0.10
		Pink	1.7	1.5	
		Pale	1.9	4.0	
		Very red	0.4	0.2	
		NA	0.6	2.1	
Vaginal mucosa pressure test (seconds)	The mucosa was pressed against the surface of another finger for 5 s, and the time until the color was regained was measured	Median	3	3	*P* = 0.46
		IQR	[3, 3]	[3, 3]	
		Range	(0–6)	(0–5)	
		NA	1.0	2.7	
Udder soreness (% sows)	Udder soreness was recorded if the sow responded with vocalization or withdrawal to the udder being touched	No soreness	97.7	93.9	*P* = 0.65
		Soreness	1.3	1.0	
		NA	1.0	5.2	
Acute udder inflammation (% sows)	Inflammation defined as swelling/reddening of one or more glands	0	95.4	52.9	*P* = 0.13
		1	4.0	1.0	
		NA	0.6	46.2	
Abnormal shape/volume of belly (% sows)	Abnormality in belly shape and volume, including a tucked up belly	Normal	99.0	94.8	*P* = 1.00
		Abnormal	0.2	0	
		NA	0.8	5.2	
Abnormalities in the head (% sows)	Head with or without asymmetry or swellings in the head	Normal	99.2	94.4	*P* = 0.56
		Asym./swel.	0.4	0.2	
		NA	0.4	3.5	
Wounds on the coronary band (% sows)	See definitions of lesions in Table 3.	None	98.7	96.0	P = 1.0000^b^
		1 or more	0.2	0.2	
		NA	1.2	3.8	

*Missing data (data not available, NA) are presented as percentage of sows. IQR, Interquartile range*.

a *For more details, see Fogsgaard et al. (11)*.

b *Analyzed with Signed Rank Test, but shown as percentage of sows. Farm: median = 0; IQR: [0, 0]; range: (0–2), Slaughter plant: median = 0; IQR: [0, 0]; range: (0–3)*.

**Table 3:** List of clinical variables, showing significant deterioration during transportation in the 522 sows transported to slaughter under commercial Danish conditions.

**Clinical measure**	**Description^**a**^**	**Unit or category**	**Farm**	**Slaughter plant**	**Statistics**
Superficial skin lesions, total (SSL_Total; frequency)	Elongated superficial skin lesions, restricted to dermis and longer than 5 cm. Counted separately for each body part (front, middle and, hind), and censored at 15 or more SSL_Total per body part	Median	0	6	*P* < 0.001
		IQR	[0, 1]	[2, 12]	
		Range	(0–45)	(0–45)	
		NA	0	4.4	
Superficial skin lesions, front (SSL_Front, frequency)	Elongated superficial skin lesions on the front, restricted to dermis and longer than 5 cm. Censored at 15 or more SSL_Front per body part	Median	0	3	*P* < 0.001
		IQR	[0, 0]	[1, 7]	
		Range	(0–15)	(0–15)	
		NA	0.4	4.4	
Wounds (frequency)	Skin lesions involving at least dermis and a diameter larger than 1 cm	Median	1	2	*P* < 0.001
		IQR	[0, 2]	[1, 3]	
		Range	(0–7)	(0–13)	
		NA	0	2.5	
Redness and swelling (frequency)	Areas with redness and swelling	Median	0	0	*P* < 0.001
		IQR	[0, 0]	[0, 1]	
		Range	(0–4)	(0–5)	
		NA	0.6	2.9	
Torn hoofs (% sows)		None	98.7	95.2	*P* = 0.01
		>1	0.6	1.7	
		NA	0.8	3.1	
Vulva lesions (% sows)	Elongated superficial skin lesion and/or wounds on the udder (see definitions above)	None	92.3	80.3	*P* < 0.001
		>1	7.3	15.5	
		NA	0.4	4.2	
Udder lesions (frequency)	Elongated superficial skin lesion and/or wounds on the udder (see definitions above)	Median	0	0	*P* < 0.05
		IQR	[0, 1]	[0, 1]	
		Range	(0–7)	(0–8)	
		NA	1.5	4.6	
Gait Score (% and score)	0: Normal gait, symmetrical limb movement	0	87.0	78.9	*P* < 0.001^b^
	1: Normal gait, but compromised movement	1	9.6	17.1	
	2: Moderately lame	2	0.8	1.3	
	3: Severely lame, one or more non-weight bearing limbs (Not fit for transportation)	3	0	0.2	
		NA	2.7	2.5	
Skin elasticity (seconds)	Quantified by pinching the skin carefully with two fingers and recording the time (seconds) until the skin has recoiled.	Median	3	3	*P* < 0.001
		IQR	[2, 3]	[3, 4]	
		Range	(1–6)	(1–9)	
		NA	2.7	3.1	
Respiration rate (frequency)	Frequency per 30 s	Median	16	18	*P* < 0.001
		IQR	[13.5, 21]	[14, 23]	
		Range	(7–42)	(6–69)	
		NA	2.7	4.4	
Respiration quality (% sows)	Forced: abdominal movement included Superficial: involving only thorax	Normal	97.9	92.2	*P* < 0.05
		Forced	0.6	1.3	
		Superfical	0.8	1.9	
		NA	0.8	4.6	

Values are given as either frequency per sow, percentage of sows or otherwise stated. Missing data (data not available, NA) are presented as percentage of sows. IQR: Interquartile range

a *For further details see Fogsgaard et al. (11)*.

b *Analyzed with Signed Rank Test, but shown as percentage of sows. Farm: Median, 0; IQR, [0, 0]; interval, (0–2), Slaughter plant: Median, 0; IQR, [0, 0]; Interval, (0–3)*.

After arrival at the slaughter plant and unloading, sows were led to a holding pen. During the walk to the holding pen, the gait of the sows was scored. The same technicians scored the gait and subsequently conducted the same clinical examination as on the farm when the sows had reached the holding pen. The sows were kept in groups of up to 15, and were typically active and engaged in aggressive interactions. Hence, heart rate measurements were not taken at the slaughter plant. Measures not able to change during the 0–8 h of transportation, e.g., body condition score, lactation status, presence of decubital ulcers, hair cover, hoof length, leg muscle volume, vulva flux, vulva smell, and the distance between floor and the lowest part of the udder were not recorded at the slaughter plant, and are described and reported in Fogsgaard et al. (11).

### Pick-Up Facility, Trucks and Loading

Eleven of the participating farms loaded the cull sows into a mobile pick-up facility, consisting of a transfer vehicle that drove to the nearest public road, where the sows waited for up to 2 h, according to Danish legislation (27) before being loaded onto the commercial truck. One farm used an on-farm pick-up facility, where the sows were assembled before being loaded unto the commercial truck.

All trucks used in the study were approved for transportation of sows and all livestock drivers were authorized to transport sows according to Danish legislation (28).

**Figure 1:**
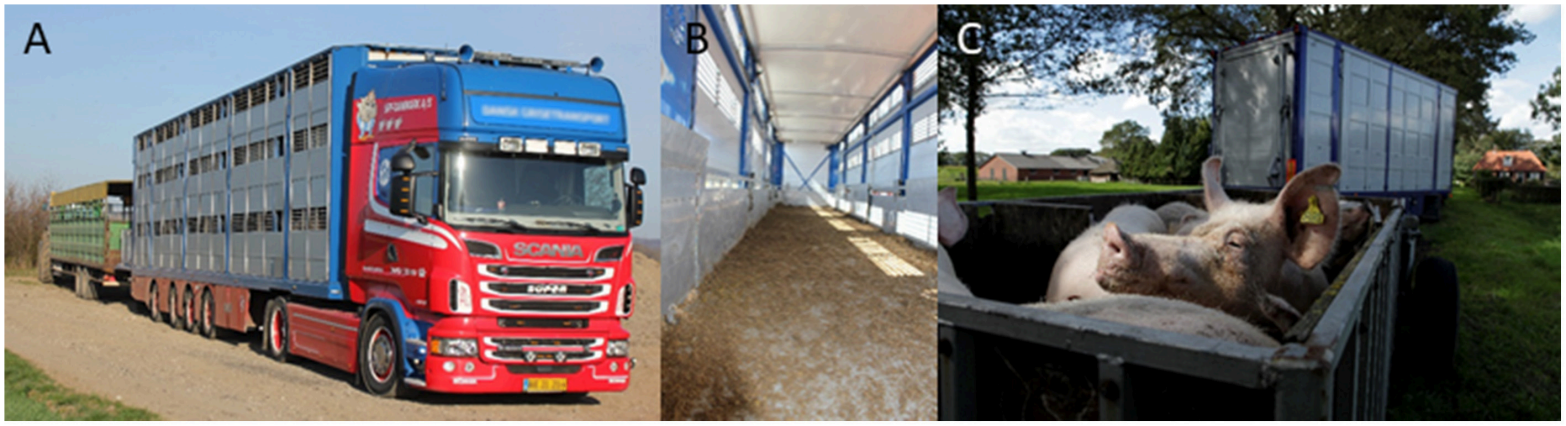
**(A)** Example of commercial truck of the type involved in the study. In the picture, the truck is parked close to the mobile pick-up facility. **(B)** The inside of the truck after removal of one deck, showing the sawdust bedding and inside walls (Credit for these two photos: Photo: Carsten Kjærulff Christensen). **(C)** Example of the type of trailers that were used in the study. The sows in the front are waiting in the mobile pick-up facility before loading (Credit Photo: Colourbox). The photos exemplify the procedures used but were not from the study.

The trucks were either single trucks, trucks with trailers or semi-trailer trucks, and had either one or two decks. Current rules regarding space allowances state that a sow with an approximate live weight of 250 kg must have 0.80 m^2^ (28). These rules were complied with at all times. In all trucks, the floors were rubber-coated and sawdust was used as bedding (Figure 1).

All ramps were coated with rubber, fitted with foot battens and the angle could be adjusted to fit the surroundings when loading and unloading sows, but was never steeper than ~26 degrees (18). The ramps were provided with side protections with an approximate height of 130–140 cm to avoid sows escaping or falling off. The trucks were both mechanically and passively ventilated through openings in the sidewalls (18). An example of the ventilation openings can be seen in Figure 1. All trucks had full air suspension. When loading, the trucks were reversed and parked close to the transfer vehicle or barn and the experimental sows were loaded directly into the truck (11.1 ± 2.8 per load; Table 4). In some cases a few sows or gilts, not part of the experiment were loaded together with the experimental sows, but this was not recorded. The driver would walk behind the sows, holding a plastic board in front of him and gently driving the sows up the ramp and onto the truck. Sticks or electric pods were never used. The loading time did not exceed ~10 min. The sows were placed on either the first or the second deck on the truck or the truck's trailer, and were sometimes split up into two groups.

**Table 4:** Information on number of sows, duration, distance, stops and temperature in this observational study of cull sow transportation.

**Variable**	**Mean**	**Std**	**N**	**Range**
Number of experimental sows per load	11.1	2.8	522	7–18
Total duration (min)	232	113	46	46-469
Distance (km)	179	101	46	29-386
Number of stops	2.4	2.3	45	0–7
Duration of stops (min)	27	32	45	0–172
Waiting time (min)	33	16	46	0–78
**MEAN TEMPERATURE (°C)**				
Total transportation	14.1	5.3	40	3.4–26.1
Until arrival	13.5	5.6	39	2.0–25.9
During waiting time	15.8	5.3	39	4.7–28.8
**MAXIMUM TEMPERATURE (°C)**				
Total transportation	18.8	4.9	40	7.6–29.1
Until arrival	16.6	5.6	39	2.6–29.1
During waiting time	18.0	5.0	39	7.6–29.1
**PERIOD WITH TEMPERATURE > 15°C (MIN)**				
Total transportation	117	127	40	0–364
Until arrival	99	114	39	0–316
During waiting time	18	20	39	0–78

*Each of the 47 loads carried sows from other farms than the ones involved in this study*.

In Denmark, the number of cull sows from one farm most often does not constitute a whole load, and hence, it is normal practice for drivers to collect sows from several farms on the way to the slaughter plant. We requested information about whether the drivers changed the sectioning, or moved sows from the truck to the trailer, or added sows to existing groups, as more sows were loaded onto the vehicle. The experimental sows were never moved during transportation, but in some cases, sows from other farms were added to an unfilled section. Hence, it was not possible to collect data for the precise stocking density or the occurrence of regrouping of sows after loading.

### Collection of Data From Loading and Until the Sows Were Unloaded

From each load of sows GPS data were kindly made available from Danish Crown enabling recording of: the distance (Distance, km) from the farm to the slaughter plant; the total transportation duration (Total duration, min); the number and duration of stops lasting at least 3 min (Number of stops); and the total duration of all stops (Duration of stops, min). We defined the total duration of a transportation as the interval between departure from the participating farm until the last sow was unloaded at the slaughter plant. In addition, the GPS data allowed calculation of the interval from departure until arrival at the slaughter plant (Duration until arrival, min), and from arrival until unloading of all sows on the truck was completed (Waiting time, min). However, as the trucks also collected cull sows from non-participating farms, the waiting time might have been overestimated, for some loads of sows, especially if the sows from the study were unloaded first, as the waiting time by definition ended when all sows on the truck had been unloaded.

Before departure from each farm, we placed a temperature logger (iButton DS1923, Maxim Integrated, San Jose, CA; resolution ≤ 0.5°C) in the compartment of the truck or trailer with the experimental sows. The loggers were placed in the middle of the truck ~0.9 m above deck level, and they recorded the temperature every minute from departure and until unloading was completed.

### Statistics

In four cases, we calculated new composite variables for further analysis based on the original clinical measures (as shown in Table 5). In addition, the total duration; duration of stops; mean temperature until arrival at the slaughter plant; and mean temperature during waiting were categorized. This was considered necessary, as the relationship between the response variables and the continuous explanatory variables (covariates) were not always linear, and hence it was needed to include these as categorical ordinal variables.

**Table 5:** Four new composite variables and four categorical constructs made from the continuous variables obtained during transportation of 522 cull sows to slaughter under commercial Danish conditions.

**Variable**	**Categories**	**Definition**
**TOTAL TRANSPORTATION DURATION**		
	TTD_1_	0–150 min
	TTD_2_	>150–260 min
	TTD_3_	>260–340 min
	TTD_4_	>340
**TOTAL DURATION OF STOPS**		
	TDP_0_	0 min
	TDP_1_	>0–30 min
	TDP_2_	>30 min
**MEAN TEMPERATURE UNTIL ARRIVAL**		
	MT_UA1_	0–10 °C
	MT_UA2_	>10–14 °C
	MT_UA3_	>14–18.4 °C
	MT_UA4_	>18.4 °C
**MEAN TEMPERATURE DURING WAITING**		
	MT_DW1_	0-12.5°C
	MT_DW2_	>12.5-15.5 °C
	MT_DW3_	>15.5-19.5 °C
	MT_DW4_	>19.5 °C
**PARITY**		
	1	Parity 1–2
	2	Parity 3–5
	3	> Parity 5
**BODY CONDITION SCORE**		
	BCS_1_	2–2.5
	BCS_2_	3–3.5
	BCS_3_	4–4.5
**VULVA CONDITION**		
	0	Normal condition
	1	Either lesion, flux, or smell
**HOOF CONDITION**		
	0	Normal condition
	1	Either long or torn hoofs or accessory digits

All data were analyzed using the SAS 9.3 software (SAS Institute Inc., Cary, NC, USA). To analyze whether measures taken before and after transportation changed, we used either a paired *t*-test (PROC Univariate), if the variables followed a normal distribution, a Signed Rank Test for ordinal variables (PROC Univariate), or a McNemar test (PROC Freq) for nominal variables. If a nominal variable had more than two outcomes, we tested the normal outcome against the alternatives. The pre- and post-values of all measures are presented as either mean ± SD (normally distributed variables); medians and interquartile range (IQR; ordinal and non-normally distributed variables); or as proportions of sows with different outcomes (nominal variables).

For a selection of the variables (superficial skin lesions, total (SSL_Total); superficial skin lesions, front (SSL_Front); wounds, skin elasticity, and gait score), we analyzed the effects of sow- and transport-related factors on the post-transportation condition. This selection was based on the variance and the size of numeric change from pre- to post-transportation of these variables.

Due to the large number of explanatory variables, the analysis was done in three steps. In the first step, we identified all explanatory variables affecting the deterioration of a response variable in a generalized linear mixed model with a binary distribution (deterioration/no deterioration) and a logit link function (PROC Glimmix). In these simple models, sow- or transport-related factors were, one by one, included as the only explanatory factor (either continuous or categorical) apart from the pre-transportation measure of the response variable, and the transportation date was included as a random factor. As we only visited one farm per day, the date also described the variation between farms. The sow- or transportation-related factors with a *P*-value of maximum 0.05 in at least one of the analyses were used as explanatory variables in the next step of the analysis.

In step two, we set up two new generalized linear mixed models, (PROC Glimmix) and with the same features as in step 1; one with sow-related and one with transportation-related factors. We used two models, due the large number of explanatory variables. In both models the pre-transportation measure of the response variable was added as an explanatory variable and in the transportation-factor model, we added explanatory variables describing the temperature during transportation. Both models were reduced by backwards reduction, removing factors and interactions when the *P*-value exceeded 0.05. However, the pre-transportation values were always kept in the model. All factors with a significant effect in at least one of the models were included in the final model.

In step three, we set up the final generalized linear mixed model with a combination of both sow and transport related factors, (PROC Glimmix and with the same features as in step 1, excluding transport distance as this was highly correlated to transport duration. The categorized explanatory variables were: total transportation duration (categorical variable used for the response variables: skin elasticity and gait score); mean temperature until arrival (categorical); the interactions between total transportation duration and mean temperature until arrival; the mean temperature while waiting (categorical); the interaction between waiting time and mean temperature while waiting; duration of stops (categorical); parity (categorical); body condition score (categorical); lactation status (yes or no); pre-transportation gait score (0–3); vulva condition (categorical); and hoof condition (categorical). Continuous explanatory variables were: total duration (for response variables SSL_Total, SSL_Front and wounds; 46–469 min); waiting time (0–78 min); number of stops (0–7); maximum temperature (7.6–29.1°C); number of minutes > 15°C (0–364); standard deviation of the temperature (0.8–3.9°C); the sow's pre-transportation body temperature (33.2–40.4°C); and the pre-transportation level of the response variable analyzed. The pre-transportation gait score was, as the only response variable, an explanatory variable in all models. We included the interaction between total duration and mean temperature before arrival, and the interaction between waiting time and mean temperature while waiting, regardless of whether the categorical or the continuous explanatory variables were used or not. Transportation date was included as a random factor.

The initial model was reduced by backwards reduction, removing factors and interactions when *P*-values exceeded 0.05. However, the pre-transportation response values were always kept in the model. To evaluate the appropriateness of the models, we used the dispersion parameter (Pearson chi-square/DF), which optimally should be close to one, and ranged from 0.98 to 1.07 in our final models. Results are presented as odds ratios (LSMEANS with 95% confidence intervals). In three cases, when a significant interaction between a continuous time variable and the mean temperature during transportation or waiting was found, we present the odds estimated for different points in time, as well as the predicted probabilities estimated by the model. In these cases, we examined exponentiated pair-wise differences between slopes in different temperature categories as a function of time, to test for differences in odds over time, e.g., the effect of increasing transportation duration or waiting time before unloading.

## Results

The 12 participating farms had an average size of 729 adult swine (range: 410–1400), including sows, gilts and boars, and delivered cull sows for slaughter every, or every second week. A total of 522 cull sows (median parity = 5; range 1–11) from the 12 farms were included in the study. We were not able to obtain information from the farms for all sows, but the available farm data showed that most culling reasons as reported by the farmers were related to reproduction, age or health. The majority of the sows were crated when we examined them in their home pen on the day of transport (Table 1). Almost 70% of the sows had a body condition score of 3 or 3.5, and close to 40% were lactating on the day of transportation (11). Sixty (11.5%) of the sows had at least one shoulder ulcer, and one sow had an umbilical outpouching. Before departure from the farms, the sows had a mean heart rate of 75 ± 18 beats per minute (11) and of the 302 sows that were lying down when the clinical examination started only nine (3%) had difficulties getting up. More than eight out of ten sows had normal hair cover; normal leg muscle volume; normal hoof and accessory digit length; and normal vulva flux with no smell [see (11) for further details].

A total of 47 loads of cull sows were included in the study. The average number of loads from each of the involved farms was 3.9 (median: 5; IQR:1.5; 5; range: 1–6) and three of the farms contributed with only one load. Four sows were considered unfit for transport after the pre-transportation clinical examination -on the farm (one with a large shoulder ulcer, one with a rectal temperature above 40.5°C, and two severely lame sows), and these were not included in the study. Three sows arrived at the slaughter plant in a condition where they, according to the current EU legislation (18), would have been judged as unfit for transport. Of these, one sow was unable to walk, the other two showed signs of fatigue and staggered, hyperventilated and were close to collapsing. All three were euthanized on the ramp, and hence no post-transportation data are available.

### Transportation Data

The mean total duration of transportation was 232 ± 113 min, and a mean distance of 179 ± 101 km was traveled. The mean temperature in the truck, from departure and until the last sow had been unloaded, was 14.1 ± 5.3°C ranging from 3.4 to 26.1°C (See details in Table 4).

### Clinical Examination at the Slaughter Plant

Approximately half of the clinical variables that were recorded before and after transportation did not change significantly. Among these were the sows' general condition, body temperature, color of mucosa and measures related to udder health. A list of these variables are shown in Table 2.

The clinical variables that changed during transportation comprised mainly injuries and measures possibly related to heat stress (Table 3). The condition of the sows deteriorated in terms of an increased number of superficial skin lesions per sow, on the front (*P* < 0.001) as well as in total (*P* < 0.001). Upon arrival at the slaughter plant, the sows had more wounds (*P* < 0.001), more swellings and reddened body areas (*P* < 0.001). The number of sows with at least one hoof torn off or vulva lesions had increased (*P* < 0.05 and *P* < 0.001, respectively). The gait score of the sows deteriorated (*P* < 0.001), but very few sows (0.2%) were severely lame at arrival to the slaughter plant. The sows had a higher respiratory rate (*P* < 0.001), and more sows showed signs of forced or superficial respiration (*P* < 0.05) than before transportation. Skin elasticity was reduced upon arrival, indicating an increased degree of dehydration (*P* < 0.001).

As expected, the number of shoulder ulcers did not increase, but shoulder ulcer characteristics did change (Table 6). We recorded more reddening in and around the ulcers (*P* = 0.01 and *P* < 0.01, respectively).

**Table 6:** The number sows with wounds and shoulder ulcers and the characteristics of the two types of lesions before and after transportation for 522 cull sows transported to slaughter under commercial Danish conditions.

	**Farm**		**Slaughter plant**		**Statistics**
**WOUNDS**					
Sows with ≥ 1 wounds	285 (54.6%)		382 (75.1%)		See Table 3
NA	0 (0%)		13 (2.5%)		
**AMONG THE SOWS WITH WOUNDS:**	**(*****n*** **= 283)**		**(*****n*****=382)**		
**No. of wounds per sow with:**	**Median (range)**	**Mean**	**Median (range)**	**Mean**	
Crust	1 (0–4)	0.85	0 (0–5)	0.64	NS
Bleeding	0 (0–5)	0.40	1 (0–13)	1.54	*P* < 0.001
Redness	0 (0–3)	0.05	0 (0–3)	0.22	*P* < 0.001
Swelling	1 (0–5)	0.71	0 (0–6)	0.52	NS
Flux	0 (0–1)	0.02	0 (0–1)	0.01	NS
**SHOULDER ULCERS**^**a**^					
Sows with 1 shoulder ulcer	47 (9.0%)		47 (9.0%)		
Sows with 2 shoulder ulcers	13 (2.5%)		13 (2.5%)		
Sows without	462 (88.5%)		462 (88.5%)		
**AMONG THE SOWS WITH SHOULDER ULCERS:**					
**No. of shoulder ulcers per sow with:**	**Median (range)**	**Mean**	**Median (range)**	**Mean**	
Crust	1 (0–2)	0.93	1 (0–2)	0.87	NS
Redness	0 (0–2)	0.26	0 (0–2)	0.50	*P* = 0.01
Redness at border	0 (0–2)	0.25	0 (0–2)	0.45	*P* < 0.01
Swelling	0 (0–2)	0.22	0 (0–2)	0.22	NS
Asymmetry of the shoulders	0 (0–1)	0.03	0 (0–1)	0.10	NS
Diameter of ulcer (cm)^b^		2.9 ± 1.3		2.7 ± 1.5	NS

*Data for wound characteristics were not normally distributed, but for clarity, we, in addition to medians and range, also present the results as means per sow. Missing data (data not available, NA) are presented as number and percentage of sows*.

a *No missing observations*.

b *Mean ± SD*.

After transportation, 382 sows had at least one extra wound (excluding shoulder ulcers), compared to 285 sows pre-transportation (Tables 3 and 6), and when we compared the characteristics of wounds per sow pre- and post-transportation, we found that a larger proportion of the wounds were bleeding and red after transportation than before (Table 6).

## Effects of Sow- and Transportation-Related Factors on the Clinical Condition of the Cull Sows Upon Arrival at the Slaughter Plant

### Superficial Skin Lesions, Total (SSL_Total)

**Figure 2:**
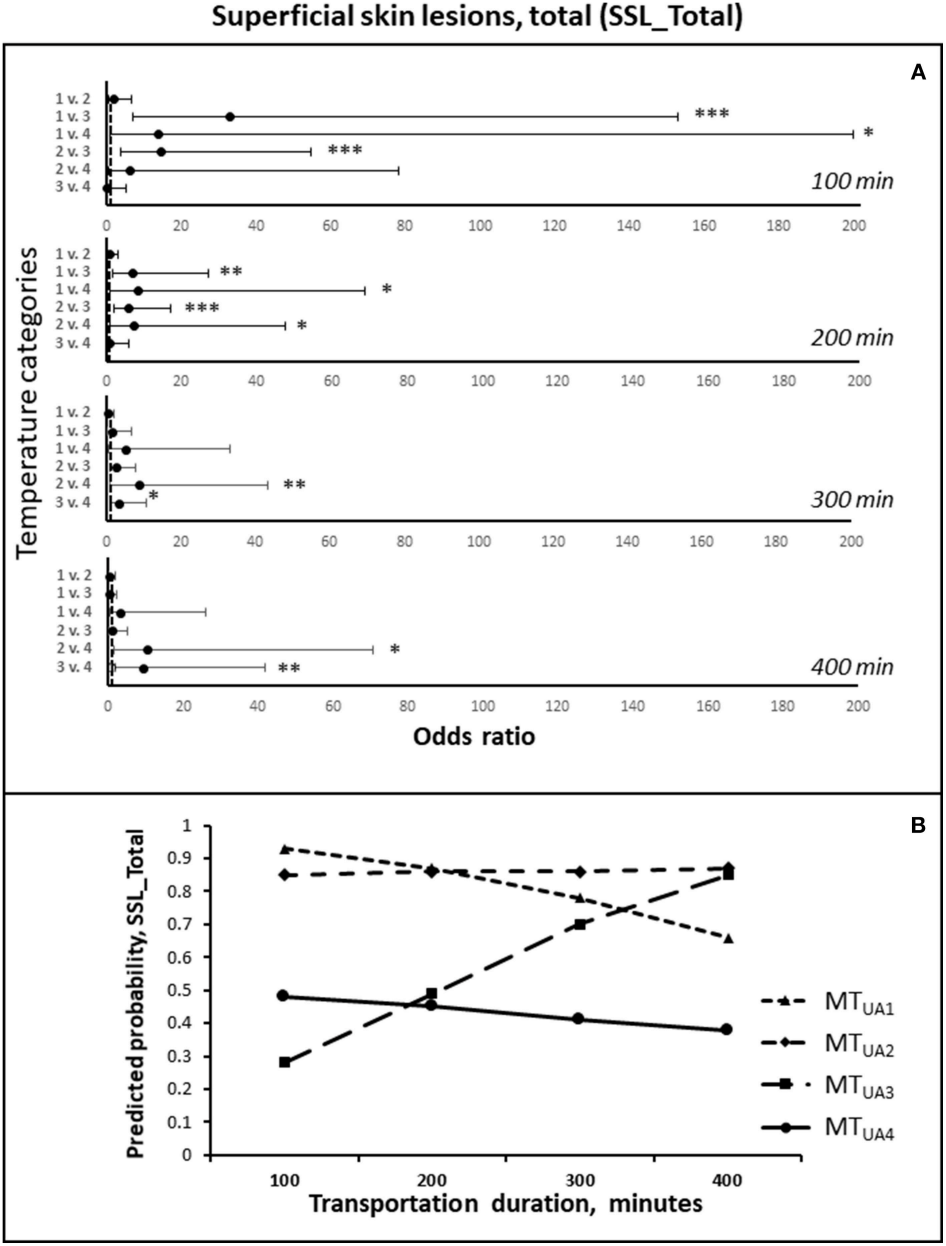
**(A)** The odds ratios (LSMEANS and 95% confidence intervals) of getting more superficial skin lesion during transportation, in total (SSL_Total), comparing the four temperature intervals, at estimated transportation durations of 100, 200, 300, and 400 min, respectively. The odds ratios are shown on the X-axis, and the numbers 1–4 on Y-axis represent the four temperature intervals: 1 = MT_UA1_ (0–10°C), 2 = MT_UA2_ (>10–14°C), 3 = MT_UA3_ (>14–18.4°C), 4 = MT_UA4_ (>18.4°C) (Table 5). **(B)** The predicted probabilities of getting more SSL_Total during transportation for each of the four temperature intervals, estimated for transportation durations of 100, 200, 300, and 400 min, respectively. * *P* < 0.05; ** *P* < 0.01, *** *P* < 0.001.

The risk of getting an increased number of superficial skin lesions (SSL_Total) was affected by duration of transportation in interaction with the mean temperature in the truck from loading and until arrival at the slaughter plant [*F*_(3, 410)_ = 6.35; *P* < 0.001]. The estimated odds ratios for the six comparisons of the four temperature intervals at durations of 100, 200, 300, and 400 min are presented in Figure 2A. In Figure 2B, the predicted probabilities have been inserted in order to provide an overview of the effect of the duration of transportation. At the shortest duration estimate, a low mean temperature in the truck led to higher odds for arriving with more SSL_Total, whereas for the longest transportation durations, intermediate mean temperatures from 10 to18.4°C (MT_UA2_ and MT_UA3_), resulted in the highest odds for an increased number of superficial skin lesions.

The development in the odds with increasing duration differed between the four temperature intervals. The odds of increased number of SSL_Total increased with time at mean temperatures from 10 to 14°C (MT_UA2)_ compared to at temperatures below 10°C (MT_UA1_; *P* < 0.05). If the mean temperature was from 14 to 18.4°C (MT_UA3_), the odds increased with longer duration of transportation compared to MT_UA2_ (*P* = 0.01) and MT_UA1_ (*P* < 0.001), and tended to increase more as compared to when the mean temperatures were above 18.4°C (MT_UA4_; *P* = 0.06).

The odds of an increased number of SSL_Total increased with increasing temperature during waiting before unloading [*F*_(3, 410)_ = 4.75; *P* < 0.01]. At temperatures above 19.5°C (MT_DW4_), the odds, compared to temperatures between 15.5 and 19.5°C (MT_DW3_), between 12.5 and 15.5°C (MT_DW2_) or 12.5°C or lower (MT_DW1_), were 5.41 (1.52–19.23; *P* = 0.01); 17.55 (3.85–76.92; *P* < 0.001) and 12.05 (1.97–71.43; *P* < 0.001) times higher, respectively. Furthermore, the odds of having an increased number of superficial skin lesions were 3.24 times higher (1.14–9.17; *P* < 0.05) at MT_DW3_, compared to at MT_DW2_.

An increase in the total duration of stops before arrival increased the odds of having an increased number of SSL_Total [*F*_(2, 410)_ = 5.97; *P* < 0.01; Table 7]. For non-lactating sows the odds of an increased number of SSL_Total were 2.53 times higher (1.40–4.56) compared to lactating sows [*F*_(1, 410)_ = 9.54; *P* < 0.01], and decreased with an increasing standard deviation in the temperature during transportation [*F*_(1, 410)_ = 10.80; *P* = 0.01]. The number of SSL_Total before departure affected the odds of having an increased number of SSL_Total negatively [*F*_(1, 410)_ = 16.94; *P* < 0.001].

**Table 7:** Odds for the deterioration of clinical measures from 522 cull sows transported to slaughter under commercial Danish conditions for different levels of transportation-related risk factors.

**Clinical measure**	**Risk factor**	**Level^**a**^**	**Odds**	**Limit, low**	**Limit, high**	**Statistics**
SSL_Total	Total duration of stops	TDP_1_ v. TDP_0_	3.08	1.34	7.09	*P* < 0.01
		TDP_2_ v. TDP_0_	6.10	2.18	17.24	*P* < 0.001
		TDP_2_ v. TDP_1_	1.98	0.97	4.07	ns (0.06)
SSL_Front	Total duration of stops	TDP_1_ v. TDP_0_	2.34	0.99	5.49	*P* = 0.05
		TDP_2_ v. TDP_0_	4.03	1.28	12.66	*P* < 0.05
		TDP_2_ v. TDP_1_	1.72	0.84	3.54	ns
Wounds	Total duration of stops	TDP_1_ v. TDP_0_	1.83	0.87	3.83	ns
		TDP_2_ v. TDP_0_	3.86	1.26	8.85	*P* < 0.05
		TDP_2_ v. TDP_1_	1.83	0.96	3.51	ns (0.07)
Skin elasticity	Total duration of stops	TDP_1_ v. TDP_0_	1.85	0.76	4.48	Ns
		TDP_2_ v. TDP_0_	6.67	1.87	23.81	*P* < 0.01
		TDP_2_ v. TDP_1_	3.60	1.57	8.20	*P* < 0.01
SSL_Front	Number of stops	Increase of 1 stop	0.85	0.71	1.01	ns (0.07)
Wounds	Number of stops	Increase of 1 stop	0.83	0.70	0.97	*P* < 0.05
Skin elasticity	Number of stops	Increase of 1 stop	0.71	0.58	0.86	*P* < 0.001
Skin elasticity	Mean temperature until arrival	MT_UA1_ v. MT_UA2_	3.11	1.37	7.07	*P* < 0.01
		MT_UA1_ v. MT_UA3_	10.32	2.85	37.41	*P* < 0.001
		MT_UA1_ v. MT_UA4_	10.39	2.11	51.22	*P* < 0.01
		MT_UA2_ v. MT_UA3_	3.32	1.14	9.70	*P* < 0.05
		MT_UA2_ v. MT_UA4_	3.34	0.89	12.68	ns
		MT_UA3_ v. MT_UA4_	1.01	0.44	2.32	ns
Skin elasticity	Total transportation duration	TTD_1_ v. TTD_2_	1.66	0.62	4.48	ns
		TTD_1_ v. TTD_3_	0.99	0.31	3.15	ns
		TTD_1_ v. TTD_4_	5.81	1.35	25.01	*P* < 0.05
		TTD_2_ v. TTD_3_	0.60	0.27	1.33	ns
		TTD_2_ v. TTD_4_	3.48	1.12	10.94	*P* < 0.05
		TTD_3_ v. TTD_4_	5.86	2.43	14.14	*P* < 0.001
Gait score	Total transportation duration	TTD_1_ v. TTD_2_	0.09	0.03	0.31	*P* < 0.001
		TTD_1_ v. TTD_3_	0.18	0.05	0.67	*P* = 0.01
		TTD_1_ v. TTD_4_	0.14	0.04	0.54	*P* < 0.01
		TTD_2_ v. TTD_3_	1.96	0.90	4.26	ns
		TTD_2_ v. TTD_4_	1.50	0.64	3.53	ns
		TTD_3_ v. TTD_4_	0.77	0.31	1.91	ns

a *See definitions of categories in Table 5*.

### Superficial Skin Lesions, Front (SSL, Front)

**Figure 3:**
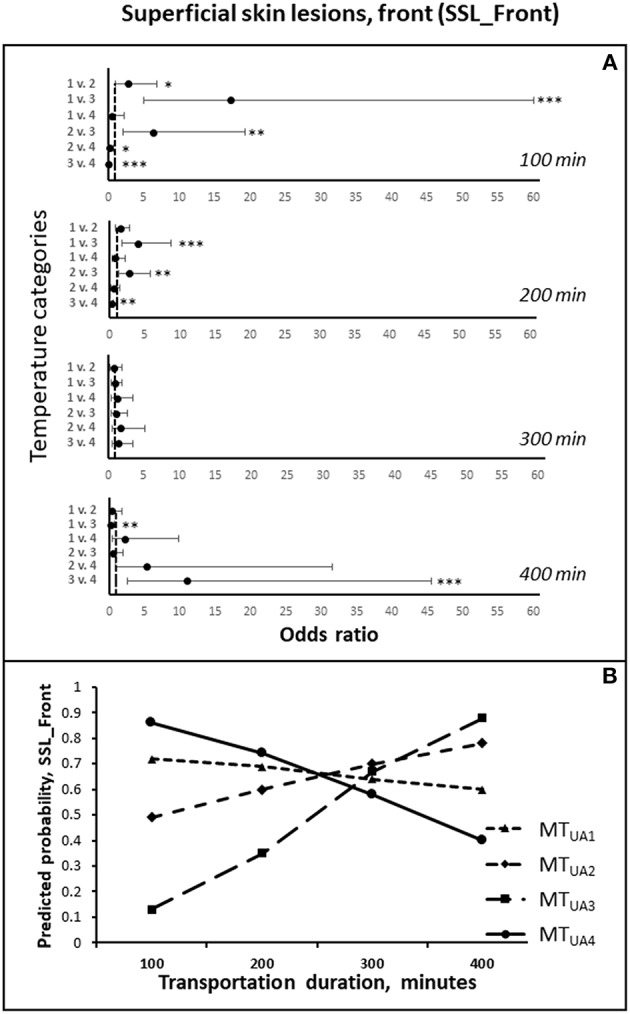
**(A)** The odds ratios (LSMEANS and 95% confidence intervals) of getting more superficial skin lesion on the front during transportation (SSL_Front), comparing the four temperature intervals, at estimated transportation durations of 100, 200, 300, and 400 min, respectively. The odds ratios are shown on the X-axis, and the numbers 1–4 on Y-axis represent the four temperature intervals: 1 = MT_UA1_ (0–10°C), 2 = MT_UA2_ (>10–14°C), 3 = MT_UA3_ (>14–18.4°C), 4 = MT_UA4_ (>18.4°C) (Table 5). **(B)** The predicted probabilities of getting more SSL_Front during transportation for each of the four temperature intervals, estimated for transportation durations of 100, 200, 300, and 400 min, respectively. * *P* < 0.05; ** *P* < 0.01, *** *P* < 0.001.

The risk of having an increased number of superficial skin lesions on the front of the body (SSL_Front) upon arrival at the slaughter plant was affected by the duration of transportation in interaction with the mean temperature in the trucks from loading and until arrival at the slaughter plant [*F*_(3, 410)_ = 8.25; *P* < 0.001; Figures 3A,B]. At shorter durations, the odds of an increase in SSL_Front were lower at 14 to 18.4°C (MT_UA3_) compared to all other temperature intervals. When the mean temperature was from 10 to 14°C (MT_UA2_), the odds of having an increased number of SSL_Front were lower compared to above 18.4°C (MT_UA4_) and below 10°C (MT_UA1_). At the longest transportation durations, the odds were highest at MT_UA3_ compared to temperatures in the intervals MT_UA4_ and MT_UA1_.

The development in the odds with increasing duration of transportation differed between the four temperature intervals, and the odds increased with time at MT_UA3_ compared with all other temperature interval (MT_UA1_: *P* < 0.001; MT_UA2_: *P* = 0.01; MT_UA4_: *P* < 0.001). Furthermore, the odds increased the longer the transportation duration at MT_UA2_ as compared to at MT_UA4_ (*P* < 0.05).

An increase in the total duration of stops before arrival tended to increase the odds of having more SSL_Front [*F*_(2, 410)_ = 2.87; *P* = 0.06; Table 7], whereas the risk tended to decrease with an increasing number of stops [*F*_(1, 410)_ = 3.29; *P* = 0.07; Table 7]. For non-lactating sows the odds of increased SSL_Front were 2.22 times higher (1.29–3.87), compared to lactating sows [*F*_(1, 410)_ = 8.25; *P* < 0.05]. The number of SSL_Front while still on the farm affected the odds of having an increased number of SSL_Front upon arrival, negatively [*F*_(1, 410)_ = 19.59; *P* < 0.001].

### Other Wounds Than Shoulder Ulcers

**Figure 4:**
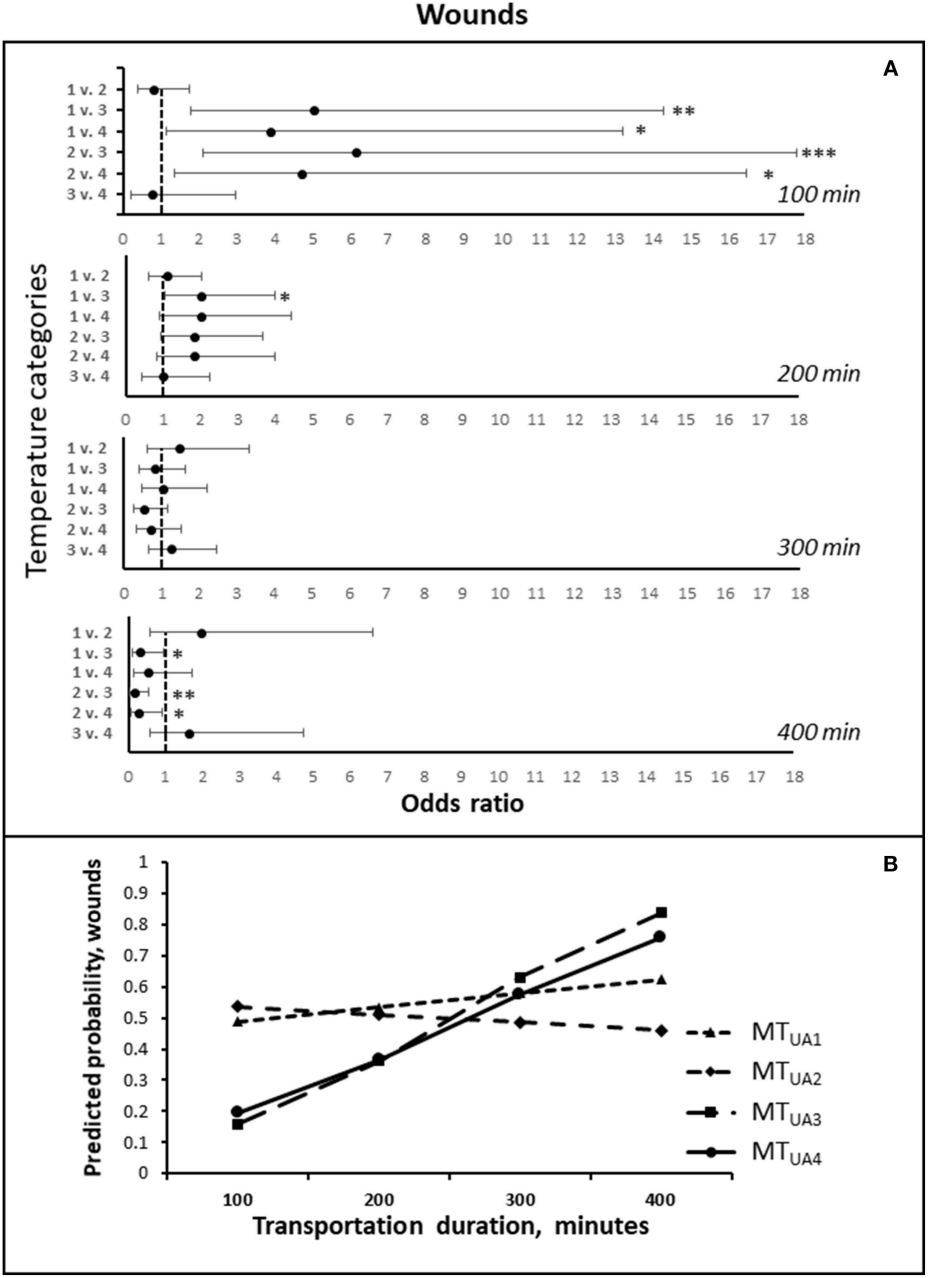
**(A)** The odds ratios (LSMEANS and 95% confidence intervals) of getting more wounds during transportation, comparing the four temperature intervals, at estimated transportation durations of 100, 200, 300, and 400 min, respectively. The odds ratios are shown on the X-axis, and the numbers 1–4 on Y-axis represent the four temperature intervals: 1 = MT_UA1_ (0–10°C), 2 = MT_UA2_ MT_UA2_ (>10–14°C), 3 = MT_UA3_ (>14–18.4°C), 4 = MT_UA4_ (>18.4°C) (Table 5). **(B)** The predicted probabilities of getting more wounds during transportation for each of the four temperature intervals, estimated for transportation durations of 100, 200, 300, and 400 min, respectively. * *P* < 0.05; ** *P* < 0.01, *** *P* < 0.001.

The odds of having a higher number of wounds after transportation were affected by an interaction between the transportation duration and the mean temperature in the trucks until arrival at the slaughter plant [*F*_(3, 402)_ = 5.89; *P* < 0.001; Figures 4A,B]. If the transportation duration was low (at the 100 min estimate), the odds of getting more wounds were significantly higher at temperatures up to 14°C (MT_UA1_ + MT_UA2_) compared to temperatures above 14°C (MT_UA3_). At intermediate durations (200 and 300 min), the temperature influenced the odds to a lesser extent, but at 200 min temperatures below 10°C doubled the odds of getting more wounds compared to MT_UA3_. In the longest transportation durations, high temperatures increased the odds, and at temperatures above 18.4°C (MT_UA4_), the odds were 3.68 times higher, compared to MT_UA2_. When temperatures were in MT_UA3_, the odds were 3.10 and 6.02 higher compared to at MT_UA1_ and MT_UA2_.

The development in the odds with increasing transportation duration differed between the four temperature intervals. The odds increased with time at mean temperatures from 14 to 18.4°C as compared to mean temperatures below 10°C (*P* < 0.01), and from 10 to 14°C (*P* < 0.05). Likewise, the odds increased if the mean temperature was above 18.4°C as compared to temperatures in MT_UA1_ (*P* < 0.05) and MT_UA2_ (*P* < 0.01).

An increase in the total duration of stops before arrival increased the odds of getting more wounds [*F*_(2, 402)_ = 3.03; *P* < 0.05; Table 7], whereas the risk decreased with an increasing number of stops [*F*_(1, 402)_ = 5.46; *P* < 0.05; Table 7]. A longer waiting time before unloading lowered the risk of getting more wounds and the odds were lowered by 0.79 (0.65–0.97) if the waiting time was prolonged by 10 min [*F*_(3, 402)_ = 5.40; *P* < 0.05].

The body temperature of the sows before loading affected the odds of having more wounds upon arrival [*F*_(1, 402)_ = 6.66; *P* = 0.01], and were 0.64 (0.45–0.90) for every 1°C increase of the body temperature. The number of wounds found before departure affected the odds of having more wounds upon arrival negatively [*F*_(1, 402)_ = 4.50; *P* < 0.05].

### Skin Elasticity

The probability of a reduction in skin elasticity depended on the maximum temperature in the truck [*F*_(1, 401)_ = 6.56; *P* = 0.01] and the odds of a reduced skin elasticity increased 1.9 (1.16–3.07) times for every 5°C increase in the maximum temperature. For every 15 min increase in the duration of time spent at temperatures above 15 °C, the odds were 1.15 [1.06–1.25; *F*_(1, 401)_ = 11.89; *P* < 0.001] greater than in periods with no increase. The mean temperature until arrival had the opposite effect [*F*_(3, 401)_ = 4.55; *P* < 0.01], and the odds of an increased skin elasticity were highest at lower temperatures (Table 7). The transportation duration affected the odds of an increased skin elasticity [*F*_(3, 401)_ = 5.59; *P* = 0.001; Table 7], which were lowest at the longest transportation durations.

If the truck was stationary for more than 30 min the odds of decreased skin elasticity increased compared to when there were no stops, or when the total duration of stops was between 1 and 30 min [*F*_(2, 401)_ = 5.20; *P* < 0.01; Table 7]. The odds decreased with an increasing number of stops [*F*_(1, 401)_ = 11.81; *P* < 0.001; Table 7]. If skin elasticity quantified on the farm was already low, the odds of reduction was lower, and conversely the odds of reduction were higher if skin elasticity was high at departure [*F*_(1, 401)_ = 68.12; *P* < 0.001].

### Gait of the Cull Sows

The odds of having a higher gait score were smaller in the shortest transportation duration interval [*F*_(3, 420)_ = 5.23; *P* < 0.01; Table 7]. An increase in the waiting time before unloading reduced the odds of having an increased gait score and were 0.73 (0.56–0.96) per 10 min increase in the waiting time [*F*_(1, 420)_ = 5.23; *P* < 0.05]. A large standard deviation in temperature during transportation increased the odds [*F*_(1, 420)_ = 5.82; *P* < 0.05], as did a high body temperature while on the farm [*F*_1, 420)_ = 4.59; *P* < 0.05]. The gait score quantified on the farm did not affect the post-transportation score.

## Discussion

The present study is among the first to focus on changes in the clinical condition of cull sows after transportation to slaughter. Based on on-farm recordings of clinical measures and repeated measurements upon arrival at the slaughter plant, the results show that transportation led to deterioration of the clinical condition of the sows, as indicated by increased prevalence of superficial skin lesions, different types of wounds as well as signs of dehydration. Among the suggested risk factors for this deterioration are mainly conditions related to transportation such as temperature in the trucks and duration of transportation—often in interaction—as well as duration of stationary periods during the transportation or while waiting to be unloaded at the slaughter plant. Below, we discuss these findings in relation to the current practice and legislation on cull sow transportation. In addition, we discuss needs for future research and development of the pre slaughter logistic chain to enable transportation of sows to slaughter without jeopardizing their clinical condition and welfare.

The major finding from the present study was that for the studied group of cull sows, several types of injuries deteriorated significantly during transportation to slaughter. Examples of these are number of vulva lesions, udder lesions, wounds, torn hoofs and superficial skin lesions. In the European regulation for transportation of animals, it is stated that “all animals shall be transported in conditions guaranteed not to cause them injury or unnecessary suffering” (18). The present findings, including increased prevalence of injuries upon arrival at the slaughter plant, might thus be interpreted as a contravention of the legislation. However, to date the regulation does not specify any definition of “injury.” Hence, it is unclear whether the deteriorations observed in the present study and their severity would be considered as non-compliance in legal terms.

Irrespective of the underlying legislation, our results show that the welfare of cull sows can be challenged by transportation from farm to slaughter, and call for further research and development of practices in order to be able to send sows to slaughter under conditions not jeopardizing their welfare.

In this data set, only 0.8% of the 526 sows selected for transportation by the farmers, and examined before being loaded for the journey, were clearly unfit according to European legislation (18) due to either large shoulder ulcers, a fever, or lameness. The remaining 522 sows were legally fit for transport at the time of departure. However, upon arrival at the slaughter plant, up to almost 8 h later, three sows were clearly unfit, unable to walk off the truck, or close to collapsing. Despite the current lack of a scientific definition of animal suffering (29, 30), the condition of these three sows was deteriorated to a degree, where use of the legal phrase “unnecessary suffering” seems relevant.

In the analyses of risk factors, we focused on the risk of deterioration and not the risk of arriving at the slaughter plant as legally unfit for transport. However, such knowledge would be important though, as fitness for transport is not a simple construct, and livestock drivers are often in doubt when assessing fitness for transport (31). A further complication related to fitness for transport is the time gap between the farmer's evaluation of the sows and the antemortem inspection upon arrival at the slaughter plant. To be able to select cull sows for transportation that maintain fitness all the way to slaughter, it is essential for farmers to know which characteristics to look for in the sows, in order to avoid deterioration of their condition. Additionally, as the characteristics of the transportation, such as duration and weather conditions seem even more important for the condition of the sows upon arrival, farmers should take this into account as well.

The present analyses of risk factors involved characteristics of the sows, such as body condition score or parity, and characteristics related to the transportation, such as duration and temperature in the vehicles. Unexpectedly, few of the sow characteristics came out as significant risk factors for the deterioration of the clinical condition of the sows, but factors related to transportation were highly significant. However, in the current study design, it was not possible to separate all relevant factors—for example farm effects and distance to the slaughter plant—leading to a risk of confounding these, due to the lack of possibility to travel different distances from the same farms to the slaughter plant. Hence, the present results may be viewed as indications, but if they can be confirmed by use of larger, and more standardized data sets, these results suggest that the assessment of fitness for transport of cull sows can only be based on the clinical condition of the sows on-farm to some extent. The major influence seems to come from characteristics related to transportation. In such case, farmers, livestock drivers and veterinarians should focus on the planned journey when assessing whether a cull sow is expected to be able to arrive at the slaughter plant in a good condition.

Among the journey characteristics suggested as risk factors were the transportation duration—often in interaction with the temperature in the truck—the duration of stops during the journey as well as the time spent waiting in the stationary truck before being unloaded at the slaughter plant. The journey duration is an aspect of animal transportation which is central in legislation (18), often the target of public debate and NGO campaigns [e.g., (32)], and relatively easy to quantify and enforce. We are not aware of other studies that describe consequences of transportation of sows in terms of animal welfare or fitness for transportation, neither for the national Danish limit of up to 8 h (28), the European limit of 24 h (18) or limits used in other regions of the world (7). It is important to emphasize that the present results cover transportation for up to almost 8 h, but even during such relatively short journeys, the duration of time spent in the truck was—often in interaction with the temperature in the truck—a significant risk factor for deteriorations of the clinical condition of the cull sows. Based on the present results showing interactions, it is, however, likely that transportation duration as such was not the only causal factor for the described deterioration. Future studies, involving the possibility to intervene and standardize e.g., different transportation times at similar temperatures or from the same farms, are needed to clarify cause and effect underlying the present findings, and thereby to provide further evidence to support or refute the central role of the duration of transportation.

Not only the full transportation duration, but also the duration of stops (stationary periods of more than 3 min in the interval from loading until arrival at the slaughter plant), and the waiting time in the stationary truck in front of the slaughter plant, were risk factors for the deterioration of the sows' clinical condition. For these, the present study showed considerable variation (e.g., stops ranging from 0 to 172 min and waiting from 0 to 78 min). The relatively large proportion of time spent stationary also become evident when comparing the mean distance traveled (179 km) and the mean duration of the journeys (232 min). These suggest that the total journey duration for these relatively short distances was substantial, due to the collection of sows from more than one farm per journey, as well as the rest periods of the drivers. It is, however, important to emphasize that these numbers come from a relatively small data set containing only 47 journeys, stratified according to the distance from farm to slaughter plant, and thus are probably not representative. Further understanding of the influence of time spent stationary requires the collection of larger data sets, e.g., from slaughter plant databases maintained as part of the optimization of the logistics [as described by (33)].

As mentioned, the stationary periods covered both collection of sows from more than one farm per journey and the statutory rest periods of the drivers (18). Drivers must rest for 45 min after each 4.5 h driving bout. At present, it is not known how large a proportion of sows transported to slaughter are actually experiencing driver rest periods while in the vehicle. Since the behavior of sows while on the trucks have not been described, the current suggestion of stops being a risk factor for superficial skin lesions, wounds and signs of dehydration, cannot be fully explained. One possibility, often reported by animal transportation professionals, is that the level of inter-sow aggression increases in a stationary vehicle, and this would likely lead to the present outcomes. If future examinations—without confounding between farm, stationary periods and journey duration—can confirm that the time spent stationary is a risk factor for deterioration of the clinical condition of the sows, it calls for reconsideration of changing the legal requirements. In other types of transportation (such a fresh milk transported from farm to dairy or fallen stock transported to the processing plant), the drivers are exempted from the current rules regarding statutory rest periods (34).

Temperature in the trucks came out as risk factor for several of the clinical deteriorations. In the current data set, the mean temperature in the trucks during transportation was 14.1°C (the mean ranged from 3.4 to 26.1°C). The maximum temperature ranged from 7.6 to 29.1°C and the duration of time spent with temperatures > 15°C was on average 117 min (ranging from 0 to 364 min). Even though these results only marginally, and only in the lower range, deviate from the European recommendations for animal transportation (5–30°C) (18), the sows in the present study relatively often, and for relatively long periods of time, experienced temperatures above the thermoneutral zone, which for sows is in the range of 15–20°C (35, 36). The possibility for thermal stress becomes even more relevant because 39.3% of the sows were lactating, and thus maintaining a higher heat production than dry sows [e.g., (24, 25)], and because they were modern prolific breeds, also characterized by high heat production (19). Thus, experiencing temperatures above 15–20°C, which these sows did for ~2 h on average, may have induced thermal stress (37, 38), due to the fact that pigs lack sweat glands and consequently need to thermoregulate behaviorally (39), the possibilities of which may be limited during transportation. In recent years, the sensitivity toward heat stress has received increased scientific attention in lactating (23) and pregnant (40) sows, but heat sensitivity of cull sows has not received the same attention.

In the present study, no behavioral data were collected during transportation, and we can only speculate about the interpretation of the observed interactions between transportation duration and temperature in the trucks. However, the increase in number of superficial skin lesions [and especially lesions on front of the sows, which have been reported to be associated with aggressive behavior in pigs (41)], indicates that the level of aggression was relatively high at lower temperatures, increased with transportation duration at intermediate temperatures, and were lower (perhaps due to a general decrease in activity level) when temperatures were highest, especially in combination with long transportation durations. Recently, Herskin et al. (26) showed that the occurrence of aggressive interactions among cull sows kept in outdoor mobile pick-up facilities before being loaded onto commercial trucks, correlated positively with the temperature in the vehicles. The contradictive findings could be due to the fact that the pick-up facilities were stationary, whereas the trucks were driving most of the time and only stationary intermittently. Further research is needed in order to fully elucidate the relations between temperature in trucks transporting sows, transportation duration and the occurrence of aggression and injuries.

We found that the odds of a decrease in skin elasticity almost doubled when the maximum temperature during transportation rose by 5°C, or the longer the temperature in the trucks exceeded 15°C, which reflects the sows' challenges during high temperatures and limited opportunities to cool down by changing position and posture while in transit. Quite surprisingly, the risk of getting dehydrated was higher at the lowest mean temperatures, but combined with the findings of higher odds for superficial skin lesion at lower temperatures, it is likely that the decreased skin elasticity at low temperatures is a consequence of a higher activity level, probably due to more aggressive interactions. As the odds also increased when the total duration of stops was more than 30 min, our results suggest that the risk of being dehydrated during transportation could be attributed to periods of high temperature and be due to increased activity, probably from fighting. These possible explanations require further study, but in the short term, the results call for increased focus on management of sows during hot periods, such as avoiding transportation during the hottest hours of the day, seeking to limit the waiting time as much as possible, enabling misting of sows during stopped periods to aid evaporative cooling, or transporting sows at lower stocking densities in order to mitigate potential negative effects on animal welfare.

During the last decade, shoulder ulcers have gained increasing focus as a welfare problem in sows (42–44), especially during lactation (45). In the present study, at least one shoulder ulcer (mean diameter 3 ± 1 cm) was found in 11.5% of the sows, and two sows were declared unfit for transportation according to Danish legislation (46) while still on-farm due to ulcers exceeding five cm in diameter. Hence, in total 11.8% of the cull sows selected for transportation by the farmers had at least one shoulder ulcer. Shoulder ulcers develop due to ischemic conditions in the shoulder region while sows lie down for longer periods (42), and cannot develop during 8 h of transportation. In accordance with this, the number of shoulder ulcers observed did not differ between the clinical examinations on-farm and upon arrival at the slaughter plant. Importantly, the clinical characteristics of the lesions changed after transportation leading to increased redness and increased occurrence of bleeding. If the occurrence and severity of shoulder ulcer are part of an antemortem inspection of sows upon arrival at slaughter plants, it should be recognized that this type of ulcers may change characteristics, but not first develop, on the way from farm to slaughter.

## Conclusion

This study is the first to describe the clinical condition of cull sows before and after transportation to slaughter. Based on significant and marked changes for several of the clinical variables—such as superficial skin lesions, different types of ulcers, and signs of dehydration—these results show that the clinical condition of the cull sows deteriorated on the way from the farm to slaughter plant. These results add data to the debate on fitness for transportation in cull sows, as animals—according the European regulation—can only be transported under conditions “guaranteed not to cause them injury.” Even though the present study design did not allow full separation of single factors influencing the clinical conditions of sows, the results of the analyses of risk factors suggest that the main risk factors for the deterioration were less related to the sows, than to the characteristics of the involved journeys. Among the major suggested risk factors were duration of transportation, temperature in the trucks—often in interaction—and duration of stationary periods on the way to unloading at the slaughter plant. Future studies should focus on further identification of risk factors and on distinguishing the effects of different risk factors in order to be able to compare them and understand how they interact. This will enable the development of (management) procedures and allow transportation of cull sows to slaughter without jeopardizing their welfare.

## Author Contributions

MH conceived the initial idea. KT and KF were in charge of the data collection and statistical analyses. The manuscript was prepared and edited by all authors.

### Conflict of Interest Statement

The authors declare that the research was conducted in the absence of any commercial or financial relationships that could be construed as a potential conflict of interest.

## References

[1] Lambooij EB Transport of pigs. In: Grandin T editor. Livestock Handling and Transport. Oxfordshire: CAB International (2014). p. 280–98.

[2] Marchant-Forde JN Marchant-Forde RM Welfare of pigs during transport and slaughter. In: Marchant-Forde JN editor. The Welfare of Pigs. Animal Welfare ©Springer Science+Business Media BV (2009). p. 301–30. 10.1007/978-1-4020-8909-1_10

[3] Christensen L Barton Gade P Temperature profile in double-decker transporters and some consequences for pig welfare during transport. Occasion Publ Br Soc Anim Sci. (1999) 23:125–8.

[4] Warriss PD Brown SN Knowles TG Wilkins LJ Pope SJ Chadd SA. Comparison of the effects of fan-assisted and natural ventilation of vehicles on the welfare of pigs being transported to slaughter. Vet Rec. (2006) 158:585–8. 10.1136/vr.158.17.585 16648438

[5] Lenkaitis AC Wang X Funk TL Ellis M Murphy CM Measurements of thermal microenvironment in a swine transport trailer. In: Proceedings of the International Conference of Agricultural Engineering. Iguassu Falls City (2008).

[6] Nielsen BL Dybkjær L Herskin MS. Road transport of farm animals: effects of journey duration on animal welfare. Animal (2011) 5:415–27. 10.1017/S1751731110001989 22445408

[7] Grandin T. Transport fitness of cull sows and boars: a comparison of different guidelines on fitness for transport. Animals (2016) 6:12. 10.3390/ani6120077 27916798 PMC5187500

[8] de Jong E Appeltant R Cools A Beek J Boyen F Chiers K Slaughterhouse examination of culled sows in commercial pig herds. Livest Sci. (2014) 167:362–9. 10.1016/j.livsci.2014.07.001

[9] Zhao Y Liu X Mo D Chen Q Chen Y. Analysis of reasons for sow culling and seasonal effects on reproductive disorders in Southern China. Anim Reprod Sci. (2015) 159:191–7. 10.1016/j.anireprosci.2015.06.018 26139322

[10] OIE World Organization for Animal Health, Terrestrial Animal Health Code (2016). Available online at: http://www.oie.int/international-standard-setting/terrestrial-code/access-online/ (Accessed October, 2018).

[11] Fogsgaard KK Herskin MS Thodberg K Transportation of cull sows—A descriptive study of the clinical condition of cull sows before transportation to slaughter. Transl Anim Sci. (2018) 2:280–9. 10.1093/tas/txy057 PMC7200563 32704712

[12] Cleveland-Nielsen A Christensen G Erøsbll AK. Prevalences of welfare-related lesions at post-mortem meat-inspection in Danish sows. Prev Vet Med. (2004) 64:123–31. 10.1016/j.prevetmed.2004.05.003 15325767

[13] Knauer M Stalder KJ Karriker L Baas TJ Johnson C Serenius T. A descriptive survey of lesions from cull sows harvested at two Midwestern U.S. *facilities*. Prev Vet Med. (2007) 82:198–212. 10.1016/j.prevetmed.2007.05.017 17604857

[14] McGee M Johnson AK O' Connor AM Tapper KR Millman ST An assessment of swine marketed through buying stations and development of fitness for transport guidelines. J Anim Sci. (2016) 94:9 10.2527/msasas2016-019

[15] Malena M Voslárová E Kozák A Bělobrádek P Bedánová I Steinhauser L Comparison of mortality rates in different categories of pigs and cattle during transport for slaughter. Acta Vet. Brno (2007) 76:109–16. 10.2754/avb200776S8S109

[16] Lykke L Blaabjerg L Hartung J Investigation of Pig Transports for more than 8 Hours in Cold and Warm Weather Conditions and of the Requirements for Ventilation During the Transport. Report from Danish Meat Research Institute (2007). Available online at: www.teknologisk.dk (Accessed October, 2018).

[17] Peterson E Remmenga M Hagerman A Akkina J. Use of temperature, humidity and slaughter condemnation data to predict increases in transport losses in three classes of swine and resulting foregone revenue. Front Vet Sci. (2017) 4:67. 10.3389/fvets.2017.00067 28553641 PMC5425469

[18] Council Regulation (2005). Council Regulation (EC) No 1/2005 of 22 December 2004 on the Protection of Animals During Transport and Related Operations and Amending Directives 64/432/EEC and 93/119/EC and Regulation (EC) No 1255/97. Available online at: https://eur-lex.europa.eu/legal-content/en/TXT/?uri=CELEX%3A32005R0001 (Accessed October, 2018).

[19] Brown-Brandl TM Hayes MD Xin H Nienaber JA Li H Eigenberg RA Heat and moisture production of modern swine. Atlanta (2014) 120:469–89.

[20] de Hollander CA Knol EF Heuven HCM van Grevenhof EM Interval from last insemination to culling: II. Culling reasons from practise and the correlation with longevity. Livest. Sci. (2015) 181:25–30. 10.1016/j.livsci.2015.09.018

[21] Engblom L Lundeheim N Dalin AM Andersson K Sow removal in Swedish commercial herds. Livest Sci. (2007) 106:76–86. 10.1016/j.livsci.2006.07.002

[22] Williams AM Safranski TJ Spiers DE Eichen PA Coate EA Lucy MC. Effects of a controlled heat stress during late gestation, lactation, and after weaning on thermoregulation, metabolism, and reproduction of primiparous sows. J Anim Sci. (2013) 91:2700–14. 10.2527/jas.2012-6055 23508026

[23] Cabezon FA Schinckel AP Smith AJ Marchant-Forde JN Johnson JS Stwalley RM Technical note: initial evaluation of floor cooling on lactating sows under acute heat stress. Professional Anim Sci. (2017) 33:254–60. 10.2527/asasmw.2017.117

[24] Rosero DS van Heugten E Odle J Arellano C Boyd RD. Response of the modern lactating sow and progeny to source and level of supplemental dietary fat during high ambient temperatures. J Anim Sci. (2012) 90:2609–19. 10.2527/jas.2011-4242 22896733

[25] Jeon JH Kim DH Methods to supply chilled drinking water for lactating sows during high ambient temperatures. Ital J Anim Sci. (2014) 13:3431 10.4081/ijas.2014.3431

[26] Herskin SM Fogsgaard KK Erichsen D Bonnichsen M Gaillard C Thodberg K. Housing of cull sows in the hours before transport to the abattoir—An initial description of sow behaviour while waiting in a transfer vehicle. Animals (2017) 7:1. 10.3390/ani7010001 28025479 PMC5295151

[27] Anonymous Law No. 104, 14/02/2000, Law on Indoor Housing of Weaners Pig, Breeding and Fattening Pigs (In Danish: Lov Om Indendørshold af Smågrise, Avls- Og Slagtesvin (2000). Available online at: https://www.retsinformation.dk/forms/R0710.aspxid=773 (Accessed October, 2018).

[28] Anonymous Act No. 1729, 21/12/2006, Act on the Protection of Animal During Transport Oversættelse (In Danish: “Bekendtgørelse Om Beskyttelse af Dyr Under Transport”) (2006). Available online at: https://www.retsinformation.dk/Forms/R0710.aspx?id=2508 (Accessed October, 2018).

[29] Fordyce P Suffering in non-human animals: perspectives from animal welfare science and animal welfare law. Glob J Anim Law (2017) 5:12–53.

[30] Weary DM What is suffering in animals? In: Appleby MC Weary DM Sandoe P editors. Dilemmas in Animal Welfare. Oxfordshire: CABI Publishing (2014). p. 188–202. 10.1079/9781780642161.0188

[31] Herskin MS Hels A Anneberg I Thomsen PT. Livestock drivers' knowledge about dairy cow fitness for transport—A Danish questionnaire survey. Res Vet Sci. (2017) 113:62–6. 10.1016/j.rvsc.2017.09.008 28892662

[32] Anonymous The 8 Hours Campaign (2018). Available online at: http://www.animals-angels.com/law-politics/8hours-campaign.html (Accessed October 2018).

[33] Håkansson N Flisberg P Algers B Jonsson A Ronnqvist M Wennergren U Improvement of animal welfare by strategic analysis and logistic optimization of animal slaughter transportation. Anim Welf. (2016) 25:255–63. 10.7120/09627286.25.2.255

[34] Anonymous Act No. 328, 28/03/2007, Act on Driving Rest Periods in Road Transport. (In Danish: Bekendtgørelse on Køre-Hviletidsbestemmelserne i Vejtransport”) (2007). Available online at: https://www.retsinformation.dk/Forms/R0710.aspx?id=2606 (Accessed October, 2018).

[35] Verstegen MW Curtis SE. Energetics of sows and gilts in gestation crates in the cold. J Anim Sci. (1988) 66:2865–75. 10.2527/jas1988.66112865x 3225240

[36] Black JL Mullan BP Lorschy ML Giles LR Lactation in the sow during heat stress. Livest Prod Sci. (1993) 35:153–70. 10.1016/0301-6226(93)90188-N

[37] Wegner K Lambertz C Das G Gauly M Climatic conditions in sow barns in Northern Germany. Züchtungskunde (2014) 86:200–11.

[38] Wegner K Lambertz C Das G Reiner G Gauly M. Climatic effects on sow fertility and piglet survival under influence of a moderate climate. Animal (2014) 8:1526–33. 10.1017/S1751731114001219 24846319

[39] Ingram D. Evaporative cooling in the pig. Nature (1965) 207:415–6. 10.1038/207415a0 5885859

[40] Lucy MC Safranski TJ. Heat stress in pregnant sows: thermal responses and subsequent performance of sows and their offspring. Mol Reprod Dev. (2017) 84:946–56. 10.1002/mrd.22844 28696547

[41] Turner SP Farnworth MJ White IMS Brotherstone S Mendl M Knap P The accumulation of skin lesions and their use as a predictor of individual aggressiveness in pigs. Appl Anim Behav Sci. (2006) 96:245–59. 10.1016/j.applanim.2005.06.009

[42] Jensen HE. Investigation into the pathology of shoulder ulcerations in sows. Vet Rec. (2009) 165:171–4. 10.1136/vr.165.6.171 19666915

[43] Herskin MS Bonde MK Jørgensen E Jensen KH. Decubital shoulder ulcers in sows: a review of classification, pain and welfare consequences. Animal (2011) 5:5. 10.1017/S175173111000203X 22439998

[44] Rioja-Lang FC Seddon YM Brown JA Shoulder lesions in sows: a review of their causes, prevention, and treatment. J Swine Health Prod. (2017) 26:101–7.

[45] Zurbrigg K. Sow shoulder lesions: risk factors and treatment effects on an Ontario farm. J Anim Sci. (2006) 84:2509–14. 10.2527/jas.2005-713 16908656

[46] Anonymous The Danish Veterinary and Food Administration's Instructions for Evaluation of Shoulder Ulcers in the Welfare Inspection (In Danish: Fødevarestyrelsens Vejledning til Vurdering af Skuldersår i Forbindelse med Kontrol (2015). Available online at: https://www.foedevarestyrelsen.dk/SiteCollectionDocuments/Dyrevelfaerd%20og%20veterinaermedicin/Skulders%C3%A5rsvejledning%20-%20revideret%2017%2003%202015.pdf (Accessed October, 2018).

